# Functional interconnections of HY1 with MYC2 and HY5 in Arabidopsis seedling development

**DOI:** 10.1186/1471-2229-12-37

**Published:** 2012-03-17

**Authors:** Babu Rajendra V Prasad, Selva V Kumar, Ashis Nandi, Sudip Chattopadhyay

**Affiliations:** 1 National Institute of Plant Genome Research, New Delhi, India; 2 School of Life Sciences, Jawharlal Neheru University, New Delhi, India; 3 Department of Biotechnology, National Institute of Technology, Mahatma Gandhi Avenue, Durgapur 713209, West Bengal, India

## Abstract

Arabidopsis seedling development is controlled by many regulatory genes involved in multiple signaling pathways. The functional relationships of these genes working in multiple signaling cascades have started to be unraveled. Arabidopsis *HY1/HO1 *is a rate-limiting enzyme involved in biosynthesis of phytochrome chromophore. HY5 (a bZIP protein) promotes photomorphogenesis, however ZBF1/MYC2 (a bHLH protein) works as a negative regulator of photomorphogenic growth and light regulated gene expression. Further, MYC2 and HY1 have been shown to play important roles in jasmonic acid (JA) signaling pathways. Here, we show the genetic interactions of *HY1 *with two key transcription factor genes of light signaling, *HY5 *and *MYC2*, in Arabidopsis seedling development. Our studies reveal that although HY1 acts in an additive manner with HY5, it is epistatic to MYC2 in light-mediated seedling growth and gene expression. This study further demonstrates that HY1 additively or synergistically functions with HY5, however it works upstream to MYC2 in JA signaling pathways. Taken together, this study demonstrates the functional interrelations of HY1, MYC2 and HY5 in light and JA signaling pathways.

## Background

Light is one of the most important environmental factors for plant growth and development throughout its life cycle [1,2]. Plants have evolved with multiple photoreceptor-systems to monitor the surrounding light quality, quantity, and direction. In *Arabidopsis*, these photoreceptors include the blue/UV-A light absorbing cryptochromes (CRY1 to CRY3) and phototropins (PHOT1 and PHOT2); the red/far-red light absorbing phytochromes (phy: phyA to phyE) [3-7]. *Arabidopsis *phytochromes form homo and hetero dimers with each other [8-10]. Formation of such heteromeric photoreceptors increases the potential complexity of R/FR light sensing and signaling mechanism in plants. Similarly, light induced activation of cryptochromes leads to possible autophosphorylation and dimerization [11]. Moreover, phytochromes and cryptochromes work together either by interaction with each other in a light-dependent and interdependent manner [12,13].

*Arabidopsis *seedlings exhibit two distinct developmental patterns, photomorphogenesis or skotomorphogenesis depending on the presence or absence of light, respectively [13-15]. Skotomorphogenesis is the strategy followed under dark conditions where *Arabidopsis *seedlings exhibit elongated hypocotyl, closed cotyledons with apical hooks; whereas in presence of light, photomorphogenesis is initiated, characterized by short hypocotyl with fully developed cotyledons. This developmental change from skotomorphogenesis to photomorphogenesis is carried out by different classes of photoreceptors, and characterised by a change in the expression of about one-third of genes in the Arabidopsis genome [16-18].

Genetic screen of *Arabidopsis *seedlings for developmental defects under light conditions have led to the identification of several transcription factors that either act as a positive or negative regulator downstream to specific photoreceptor or a set of photoreceptors [19-27]. Recently, a DNA-ligand binding screen has led to the identification of three Z-box binding factors, ZBF1/MYC2, ZBF2/GBF1 and ZBF3/CAM7 [28-33]. MYC2 is a bHLH transcription factor that acts downstream to cry1 and cry2 photoreceptors, and negatively regulates blue light-mediated photomorphogenic growth and blue and far red-light regulated gene expression [29]. MYC2 also functions as a transcriptional regulator for ABA and JA signaling pathways [29,34-37].

HY5 is one of the first known and most extensively studied bZIP transcription factor involved in promoting photomorphogenesis. Arabidopsis seedlings mutant for *HY5 *exhibit elongated hypocotyl under various wavelengths of light, suggesting that functionally HY5 is downstream to multiple photoreceptors [19,38-40]. Further, *hy5 *mutant seedlings exhibit defects in root growth and reduction in chlorophyll and anthocyanin accumulation [19,41]. In addition, studies have shown the involvement of HY5 in both auxin and cytokinin signaling pathways [42-44], suggesting that HY5 might be a common intermediate in light and hormone signaling pathways. The chromatin immunoprecipitation (CHIP) assays have revealed that HY5 preferentially binds to more than 3000 chromosomal sites that were distributed in all the five chromosomes [45].

Arabidopsis *HY1 *encodes heme oxygenase (HO) that catalyses the committed step in the conversion of heme to biliveridin IXα (BV), which is further converted to photochromobilin through sequential steps and exported to cytoplasm where it binds to the newly synthesized apo-phys by an autocatalytic process to form functional holo-phytochrome. The HOs are encoded by a small gene family that includes *HY1, HO2, HO3 *and *HO4*. Among all the four members of the HO family *HY1 *is highly expressed in almost all the tissues and plays a major role in synthesis of holo-phytochrome [46]. Seedlings mutant for *HY1 *exhibits elongated hypocotyl in red and far red light, and display defects in root development. Further, the light inducible genes such as *CAB, RBCS *and *CHS *are under-expressed in *hy1 *mutant background [47-49]. Recently, it has been reported that seedlings mutant for *HY1 *show elevated levels of JA and expression of JA-inducible defense genes [50].

In this study, in order to identify genes that might be working parallel to *HY5*, a genetic screen was set up using *hy5-ks50 *mutant lines through EMS mutagenesis. Gene cloning and genetic complementation analysis revealed that one of these mutants *(enhancer of HY5: ehy5) *contains a mutation in the *HY1*gene. We have investigated the interrelations of *HY1 *with two transcription factors, HY5 and MYC2, with respect to light-controlled Arabidopsis seedling development and JA responsiveness.

## Results

### Mutations in *EHY5 *modulate HY5-controlled hypocotyl elongation

HY5 is a key transcription factor in light signaling pathways that promote photomorphogenesis under a broad spectrum of light [32,40]. Although the *hy5 *mutant seedlings display elongated hypocotyl in light, the seedlings are not completely etiolated similar to dark grown seedlings. Therefore, there might be additional factors present that are involved in the promotion of photomorphogenesis under various wavelengths of light [39]. Recent studies have shown that CAM7/ZBF3 works in various wavelengths of light to promote photomorphogenic growth and light regulated gene expression. Further, HY5 and CAM7 work synergistically or additively in the promotion of photomorphogenesis [32].

In order to find additional factors that promote photomorphogenesis in concert with HY5, an extragenic enhancer screen was set up using *hy5 *mutant lines (*hy5-ks50 *mutant; 19) through EMS mutagenesis. Several double mutant lines that showed enhanced hypocotyl growth as compared to that of *hy5 *mutants were identified. One such mutant line, *hy5 ehy5 *(*ehy5: enhancer of HY5) *double mutant, was selected for further study. The segregated *ehy5 *line (obtained in F2 population from a back cross with wild type (Ws) was repeatedly backcrossed with wild type (Ws) to purify the mutation from any other back ground mutations.

**Figure 1:**
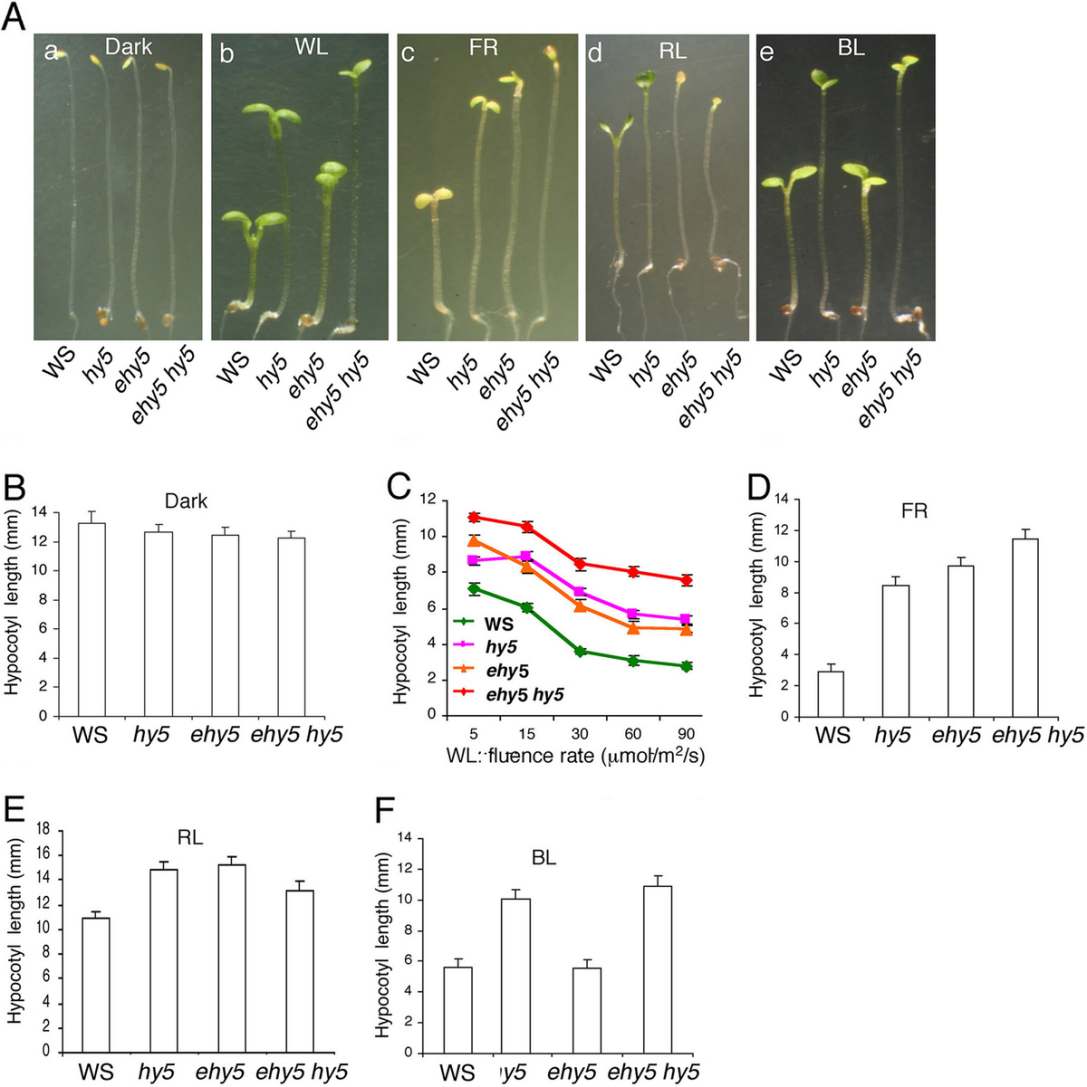
**The *ehy5 m*utants display elongated hypocotyl**. **A**, Phenotype of segregated wild-type (WS), ehy5, *hy5*, and *ehy5 hy5 *double mutants in dark and different light conditions. Six-day old constant dark, WL (90 *μ*mol m^-2 ^s^-1^), FR (90 *μ*mol m^-2 ^s^-1^), RL (90 *μ*mol m^-2 ^s^-1^) and BL (45 *μ*mol m^-2 ^s^-1^) grown seedlings (a to e). **B-C**, Quantification of hypocotyl length of 6-day-old seedlings grown in constant dark and various fluences of WL, respectively. **D-F**, Quantification of hypocotyl length of 6-day-old constant FR (90 *μ*mol m^-2 ^s^-1^), RL (90 *μ*mol m^-2 ^s^-1^) and BL (45 *μ*mol m^-2 ^s^-1^) grown seedlings, respectively. The error bar indicates standard deviation (SD). The experiment was repeated more than twice and similar results were obtained each time. A representative result is presented. For measuring hypocotyl length, ~30 seedlings were used in each genotype.

The examination of seedling morphology revealed that neither *ehy5 *alone nor *ehy5 hy5 *double mutants exhibited any altered morphology in the dark (Figure 1A (a) and 1B). The characteristic long hypocotyl phenotype of *hy5 *in WL (white light) irradiation was further enhanced in *ehy5 hy5 *double mutants, exhibiting a super tall phenotype under various fluences of WL (Figure 1A (b) and 1C). To determine whether this reduced sensitivity of *ehy5 hy5 *phenotype is specific to a particular wavelength of light, the growth of 6-day-old *ehy5 hy5 *double mutant seedlings was tested in various wavelengths of light. As shown in Figure 1A (c) and 1D, *ehy5 hy5 *double mutants displayed further reduced sensitivity to far-red light (FR) as compared to *ehy5 *and *hy5 *single mutants, suggesting that *EHY5 *and *HY5 *additively control the hypocotyl growth in FR. On the other hand, hypocotyl length of *ehy5 hy5 *double mutants was found to be closer to either of the single mutants in red light (RL), suggesting that *EHY5 *and *HY5 *are likely to work in the same branched pathways in controlling the hypocotyl length in RL (Figure 1A (d) and 1E). The *ehy5 *mutants exhibited similar hypocotyl length to that of wild type in blue light (BL), and the hypocotyl length of *ehy5 hy5 *double mutants was similar to that of *hy5 *single mutants, suggesting that additional mutation in *EHY5 *does not affect the *hy5 *phenotype in BL (Figure 1A (e) and 1F).

### Map based cloning reveals that *EHY5 *encodes HY1

**Figure 2:**
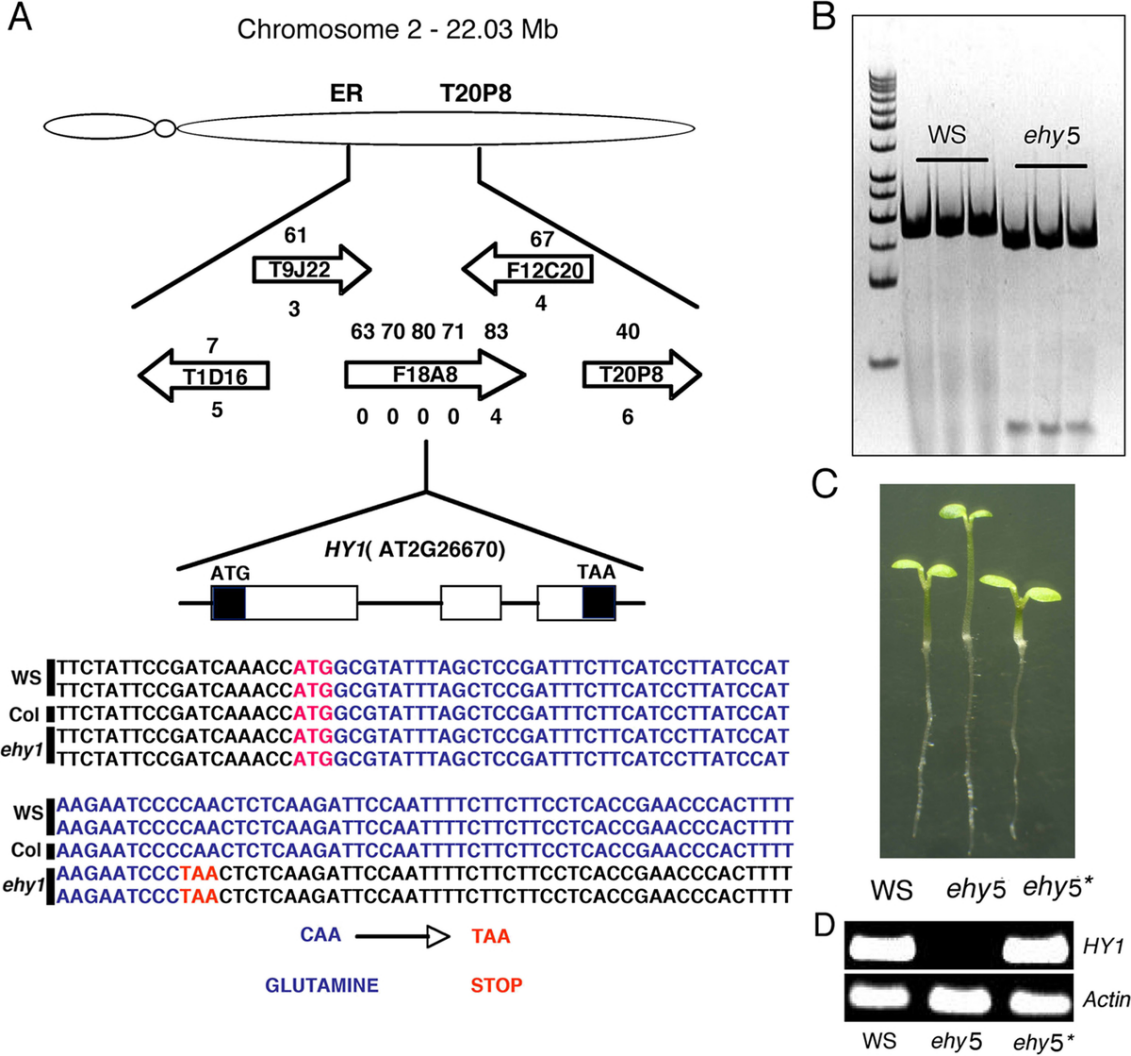
**Positional cloning and molecular identification of *EHY5***. **A**, Map-based cloning. The genetic locus of *ehy5 *mutation was first mapped between markers ER and T20P8 on chromosome 2. Fine mapping using genetic markers designed from BAC clones. The direction of the BAC clone is indicated by the arrow. The numbers above and below the arrow indicate the marker number and the corresponding recombinants for the respective marker. Sequence of the genomic DNA fragment from wild-type (WS) and *ehy5 *mutant plants and comparison with wild-type (Col) genomic DNA sequence indicate C to T mutation. **B**, DNA polymorphism between *ehy5 *and wild-type (WS) plants. The C to T mutation in *ehy5 *genomic DNA adds a *DdeI *recognition site. The DNA fragments flanking the *DdeI *site were amplified from the wild-type and *ehy5 *plants, digested with *DdeI*, and separated on native PAGE. **C**, Genetic complementation of *ehy5*. Phenotypes of 6-day-old wild-type (WS), *ehy5 *and ehy5* (*HY1 *complemented) are shown. **D**, RT- PCR results show the expression of *HY1 *in wild-type (WS), *ehy5 *and ehy5*. Actin bands show the loading control. The RT-PCR experiment was repeated thrice and a representative result is shown.

To determine the genetic basis of *EHY5 *mutation, we followed map-based cloning strategy. The *ehy5 *mutants (WS) were genetically crossed to wild type (Col), and the resulting F_1 _progeny showed wild type phenotype. F_1 _plants were self-pollinated and since the *ehy5 *long hypocotyl phenotype is easy to score at the seedling stage, the *EHY5 *locus has served as a useful landmark for classical mapping. For fine mapping, the segregating F_2 _populations with the *ehy5 *phenotype were used for mapping with Simple Sequence Length Polymorphism (SSLP) and Cleaved and Amplified Polymorphic Sequence (CAPS) markers that we developed during this study and also that are available in the database at the Arabidopsis Information Resource (TAIR). Initially, the target locus was mapped between the markers ER and T20P8 on Chromosome 2 (Figure 2A). Further fine mapping with seven genetic markers delimited the target gene to a 20-Kb region on the F18A8 BAC clone. To further identify the exact position of the EMS mutation, we have sequenced the genomic DNA fragment of the 20-Kb region from the *ehy5 *background and compared with that of wild type (WS) genomic DNA sequence, which revealed that a single C to T nucleotide substitution in the first exon of the *HY1 *(AT2G26670) DNA leads to the conversion of Glutamine (CAA) to stop codon (TAA), resulting in the premature termination of the protein translation (Figure 2A). This EMS induced substitution in *HY1 *first exon introduces a DdeI recognition site adjacent to the mutation region. We developed a dCAPS marker to confirm the mutation in *ehy5 *(Figure 2B).

As a final step to establish that the *EHY5 *locus encodes *HY1 *transcript, we tested whether a wild type genomic fragment containing the entire *HY1 *gene could complement *ehy5*. Fragment containing entire *HY1 *coding region with its native promoter was introduced into *ehy5*mutant background. As shown in Figure 2C, *ehy5 *seedlings transformed with full length *HY1 *genomic DNA fragment exhibited wild-type phenotype. The positive transformants were confirmed by RT-PCR (Figure 2D). These results indicate that the *ehy5 *mutant is an allele of *hy1 *mutant, and henceforth we refer to *ehy5 *as *hy1*.

### *HY1 *and *HY5 *additively regulate the expression of light regulated genes and accumulation of chlorophyll and anthocyanin during early seedling development

**Figure 3:**
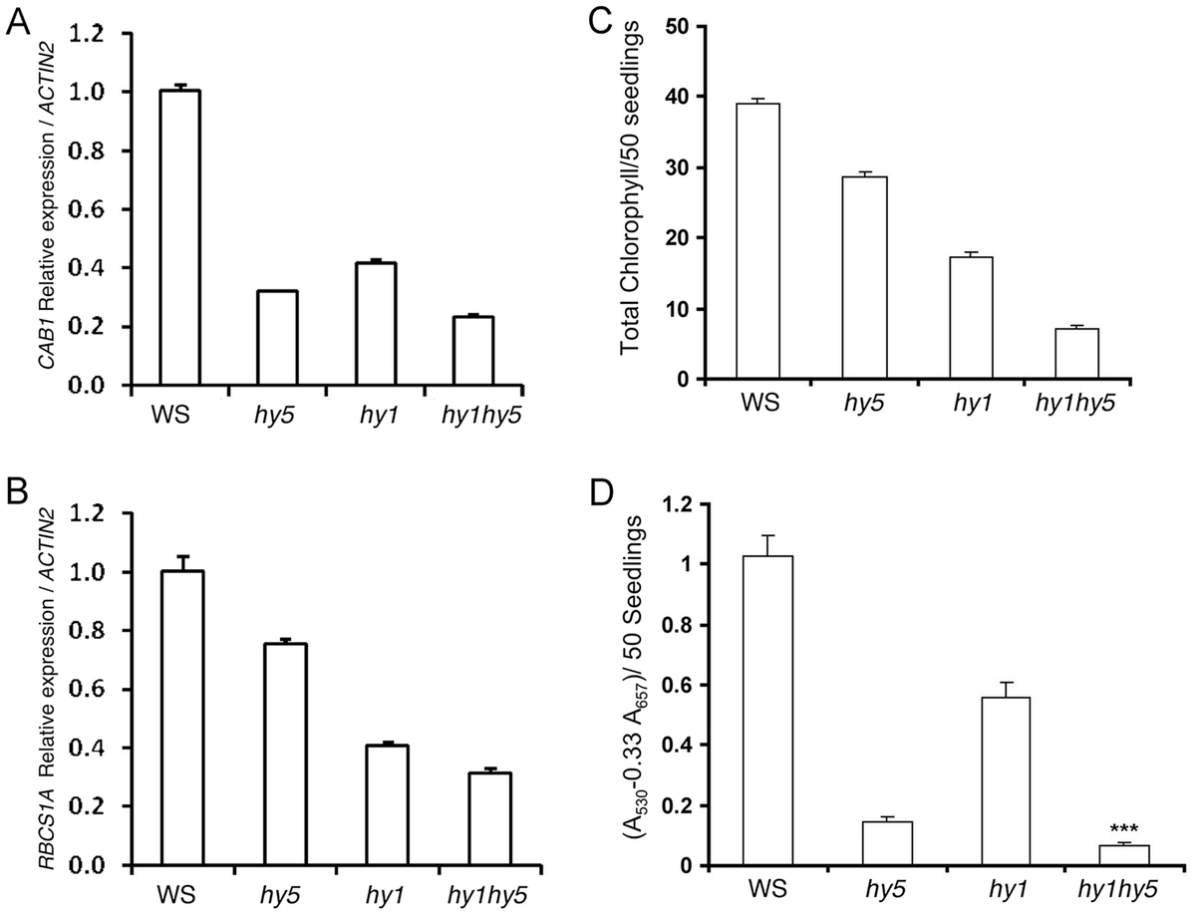
***HY1 *and *HY5 a*dditively regulate the light-induced gene expression**. **A **- **B**, Relative expression of *CAB1*and *RBCS-1A *in 6-day-old seedlings grown in WL (90 *μ*mol m^-2 ^s^-1^). **C**, Accumulation of chlorophyll in 6-day-old wild-type and mutant seedlings grown in WL (90 *μ*mol m^-2 ^s^-1^). **D**, Accumulation of anthocyanin in 6-day-old wild-type and mutant seedlings grown in WL (90 *μ*mol m^-2 ^s^-1^). The error bars indicate SD. *** - indicates significant difference from *hy5 *(p > 0.001 student's *t*-test, n = 30, number of seedlings used for hypocotyl measurement). Real-time PCR was repeated more than thrice and in each biological experiment three technical replicates were used. Similar results were obtained in all the experiments. A representative figure is shown here. For chlorophyll and anthocyanin estimation, 50 seedlings was used in each genotype and the experiment was repeated thrice and in each biological experiment, four technical replicated were used. Similar results were obtained in all the experiments. A representative figure is presented.

The loss-of-function mutants of *HY5 *display partial photomorphogenic growth at various wavelengths of light with reduced expression of light-regulated genes such as *CAB1 *and *RBCS-1A*. Similarly, *hy1 *also shows reduced accumulation of *CAB *and *RBCS *transcripts [51]. To examine how *HY5 *and *HY1 *genetically interact to regulate the expression of light inducible genes, we monitored the expression of *CAB1 *and *RBCS-1A*by real time PCR. As shown in Figure 3A-B, the expression of *CAB1 *and *RBCS-1A *was reduced in both *hy1 *and *hy5 *single mutants as compare to wild-type, and the accumulation of transcript was further reduced in *hy1 hy5 *double mutants compared to either of the single mutants. These results indicate that *HY1 *and *HY5 *act in an additive manner to regulate the expression of *CAB1 *and *RBCS-1A *genes.

Earlier studies have shown that *hy5 *and *hy1 *mutant seedlings display reduction in the accumulation of chlorophyll and anthocyanin. To determine the genetic interaction of *HY1 *and *HY5 *for chlorophyll and anthocyanin accumulation, chlorophyll and anthocyanin contents were estimated from six-day-old WL grown seedlings. As shown in Figure 3C and 3D, the *hy1 hy5 *double mutants showed less accumulation of chlorophyll and anthocyanin as compared to that of *hy1 *and *hy5 *single mutants, suggesting that HY1 and HY5 act in an additive manner to control the accumulation of chlorophyll and anthocyanin in WL.

### *HY1 *and *HY5 *work in an additive or synergistic manner to control JA responsiveness

**Figure 4:**
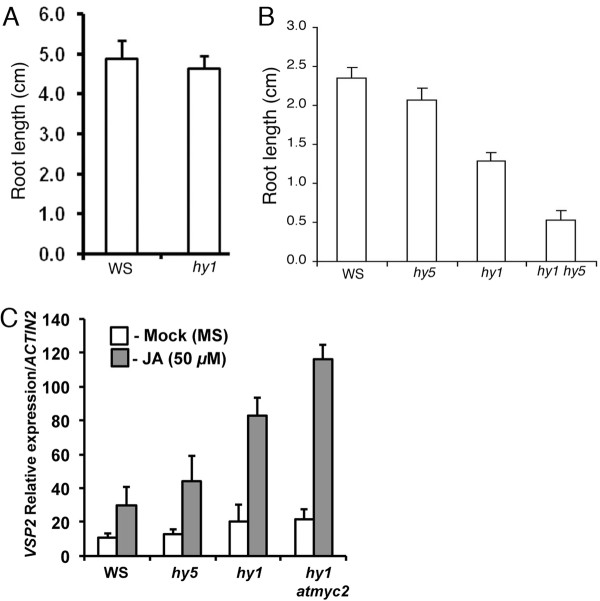
**JA responsiveness of *hy1 hy5 d*ouble mutants**. **A**, Quantification of root length of 16-day-old wild-type and *hy1 *mutant plants grown in constant WL (90 *μ*mol m^-2 ^s^-1^) without hormone (JA). **B**, Quantification of root length of A. **C**, Relative induction of *VSP2 *expression by JA in wild-type and mutant plants. Six-day-old wild-type and mutant seedlings were treated with MS (Mock) or with JA (50 *μ*M) for 5-hours and total RNA was isolated from 100 mg of tissue and used for quantitative real-time PCR analysis. *ACTIN2 *was used as internal control. Approximately 25 to 30 seedlings were used for the root growth measurement. The error bars indicate standard error (SE) of three biological replicates.

In the presence of jasmonic acid (JA), *hy1 *mutants have stunted root growth and expression of JA-inducible defence genes [50]. We asked whether mutation in *HY5 *can modulate the JA sensitiveness of *hy1 *mutants. To examine that, we grew the seedlings in the presence or absence of JA and examined the root growth. Although very little difference, if any, was observed between the wild type and *hy1 *mutants in the absence of JA (Figure 4A), 15 μM JA caused root growth retardation in *hy1 *mutant seedlings as compared to the wild type (Figure 4B). The effect was more severe in *hy1 hy5 *double mutants (Figure 4B). These results suggest a synergistic function of HY1 and HY5 in JA-mediated root growth. To determine whether the expression of JA regulated genes is affected in *hy1 hy5 *double mutants, the transcript accumulation of JA-responsive marker gene *VSP2 *was determined [35,50]. The real time PCR analyses had shown that JA treatment induced the expression of *VSP2 *both in *hy1 *and *hy5 *mutants, and the level of expression was further increased in *hy1 hy5 *background (Figure 4C). These results indicate that HY1 and HY5 function in an additive manner to regulate the expression of *VSP2 *in response to JA.

### Additional mutation in *MYC2 *abolishes the hyper-sensitive responses of *hy1 *to JA

**Figure 5:**
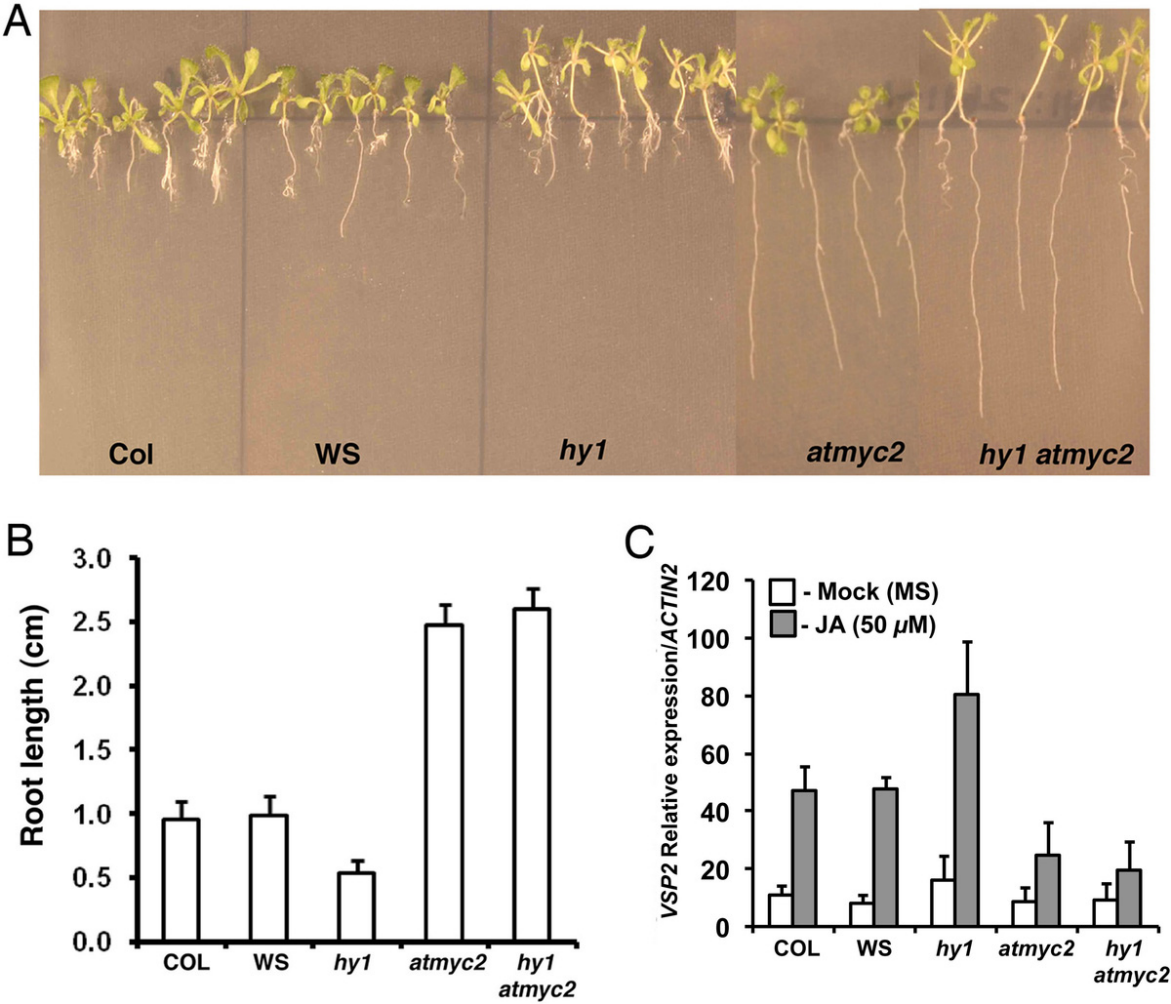
**JA responsiveness of *hy1 atmyc2 d*ouble mutants**. **A**, Root growth of 16-day-old wild-type and various mutant plants grown in constant WL (90 *μ*mol m^-2 ^s^-1^) in presence of 15 *μ*M JA. **B**, Quantification of root length of A. **C**, Relative induction of *VSP2 *expression by JA in wild-type and mutant plants. For experimental detail, see legend to Figure 4C.

MYC2, a bHLH transcription factor, acts as a negative regulator of blue light mediated photomorphogenic growth and cross talks with JA and ABA signaling pathways [29,33,36,37,52,53]. MYC2 positively regulates the expression of JA-responsive genes such as *VSP2 *by directly binding to the G-box motif present in the promoter of *VSP2 *[35,36]. Furthermore, *atmyc2 *mutant seedlings are insensitive to JA-induced inhibition of root growth. To investigate the interaction between *HY1 *and *MYC2 *with respect to JA-responses, we constructed *hy1 atmyc2 *double mutants through genetic crosses. The root growth of *hy1 atmyc2 *double mutant plants was monitored in the absence or presence of 15 μM JA. No significant difference in root length was observed among the mutants and wild type in the absence of JA. JA caused severe root growth retardation in wild-type and *hy1 *mutants, however the effect was drastically reduced in *atmyc2 *and *hy1 atmyc2 *mutant plants (Figure 5A and 5B). These results indicate that MYC2 works downstream to HY1 in JA-mediated inhibition of root growth. We then examined the expression of one of the JA-inducible marker genes *VSP2 *by real time PCR in various mutant backgrounds. As shown in Figure 5C, whereas there was very little expression of *VSP2 *in the absence of JA, the expression of *VSP2 *was increased in the presence of JA in wild-type and *hy1 *mutant plants. Further, the *hy1 *mutants showed significantly higher level of accumulation of *VSP2 *transcript as compared to wild-type background. However, the expression of *VSP2 *was less in *atmyc2 *plants, as expected from its less sensitiveness to JA, and was found to be similar to *hy1 atmyc2 *double mutants. These results suggest that MYC2 works downstream to HY1 in JA-induced expression of *VSP2 *gene.

### Overlapping functions of HY1 and MYC2 in Arabidopsis seedling development

**Figure 6:**
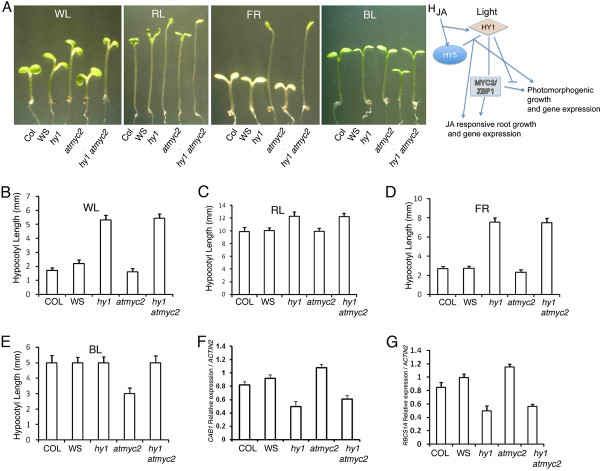
**Light-mediated seedling development of *hy1 atmyc2***. **A**, Phenotype of wild-type and various mutant seedlings in different light conditions. Six-day-old constant WL (90 *μ*mol m^-2 ^s^-1^), RL (90 *μ*mol m^-2 ^s^-1^), FR (90 *μ*mol m^-2 ^s^-1^) and BL (45 *μ*mol m^-2 ^s^-1^) grown seedlings. **B-E**, Quantification of hypocotyl length of 6-day-old constant WL (90 *μ*mol m-^2 ^s^-1^), RL (90 *μ*mol m^-2 ^s^-1^), FR (90 *μ*mol m^-2 ^s^-1^) and BL (45 *μ*mol m^-2 ^s^-1^) grown seedlings, respectively. **F-G**, The relative expression of *CAB1 *and *RBCS-1A *in 6-day-old seedlings grown in WL (90 *μ*mol m^-2 ^s^-1^). The error bars indicate SD. Approximately 25 to 30 seedlings were used for hypocotyl length measurement. For gene expression studies, total RNA was isolated from 100 mg of tissue was used for cDNA preparation. The real-time PCR experiments were repeated more than twice and three technical replicates were used for each genotype. Similar results were obtained in all the experiments. A representative graph is shown. **H**, Working model shows the role of HY1, HY5 and MYC2 in photomorphogenesis and JA responsiveness. HY1 and HY5 act additively in response to JA and light signaling pathways. MYC2 acts downstream to HY1 in JA responsiveness, and HY1 acts negatively to MYC2-mediated BL specific photomorphogenic growth.

The *atmyc2 *mutants display hypersensitive response to BL, and are epistatic to *cry1 *and *cry2 *[29]. In order to determine how these two light signaling components, HY1 and MYC2, genetically interact to control early seedling development, we measured the hypocotyl length of *atmyc2 hy1 *double mutants in various light conditions. Similar to *hy1 *or *atmyc2 *single mutants, *hy1 atmyc2 *double mutants did not show any altered growth in the dark. However, under WL conditions, *hy1 atmyc2 *double mutants displayed hypocotyl length similar to *hy1 *single mutants (Figure 6A and 6B). Furthermore, as shown in Figure 6A and 6C to 6E, *hy1 atmyc2 *double mutants displayed hypocotyl length similar to *hy1 *single mutants in RL, FR and BL conditions. These results indicate that although additional mutation in *MYC2 *does not affect the phenotype of *hy1 *mutants in RL and FR, it is able to suppress the *atmyc2 *phenotype in BL. MYC2 acts as a negative regulator of light induced gene expression such as *CAB1 *and *RBCS-1A*. We examined how the additional mutation in *MYC2 *affects the expression of light-inducible genes in *hy1 *mutant background. The real time PCR analysis revealed that the expression of *CAB1 *and *RCBS-1A *was similar to that of *hy1 *single mutant in *hy1 atmyc2 *background (Figure 6F and 6G). These results suggest that *HY1 *is epistatic to *MYC2 *in controlling the light induced gene expression.

## Discussion

Although many components of light signaling pathways are known, the interconnections of these components in Arabidopsis seedling development is unclear. Moreover, very little information is available on cross talks of various components of light signaling with other signaling cascades and vice versa. In this study, we have demonstrated the genetic interactions of *HY1 *with two other light-signaling components, HY5 and MYC2, which belong to two important families of transcription factors, bZIP and bHLH, respectively, in Arabidopsis seedling development. Furthermore, this study reveals that HY1, HY5 and MYC2 are functionally connected in JA signaling pathways.

An attempt to identify new genes that might enhance *hy5 *phenotype, similar to *CAM7/ZBF3 *led to the identification of *EHY5 *[32]. Map based cloning and genetic complementation of *ehy5 *mutants reveal that *EHY5 *codes for *HY1 (HO1)*, a rate-limiting enzyme that catalyzes the conversion of heme to biliverdin IXα (BV) in the chromophore biosynthesis pathway [54]. Phenotypic analyses under various light conditions have revealed that HY1 and HY5 function in an additive manner resulting in a super tall phenotype in WL. Similar additive function of HY5 and HY1 was also observed in the regulation of hypocotyl growth in FR. Genetic interaction studies between *HY1 *and *HY5 *reveal that they are likely to work in the same branched pathways of light signaling. On the other hand, mutations in *HY1 *does not affect the *hy5 *phenotype in BL. However, the additional mutation in *HY1 *is able to suppress the *atmyc2 *phenotype in BL. These results strongly suggest the wavelength specific interdependent functions of HY1, HY5 and MYC2 in the regulation of hypocotyl growth in Arabidopsis seedling development.

The expression of light regulated genes is down-regulated in *hy1 *mutant background. HY5 directly binds to the G-box present in the promoters of light regulated genes and promote their expression [39]. MYC2/ZBF1 also interacts with the Z-/G-box LRE present in the light-inducible promoters such as *CAB1 *and *RBCS1A*, however down-regulates their expression [29,33,45]. Analysis of light-regulated gene expression in *hy1 hy5 *double mutants reveal that HY1 and HY5 function in an additive manner and elevate the expression of light regulated genes. These two proteins also function in an additive manner to regulate the accumulation of chlorophyll and anthocyanin. On the other hand, the expression of *CAB1 *and *RBCS-1A *in *hy1 atmyc2 *double mutant seedlings was similar to that of *hy1 *single mutants, and thus suggesting that HY1 works downstream to MYC2 in the regulation of *CAB1 *and *RBCS-1A *expression. It has been shown earlier that although *atmyc2 *works downstream to cry1 and cry2 photoreceptors, *phyA *is epistatic to *atmyc2 *in BL [29].

Plant growth and development is a complex phenomenon, which is likely to be regulated through interactions between light and phytohormone signaling pathways. Recent studies have shown that signals from light and multiple hormonal signaling pathways cross talk through common downstream regulatory proteins such as MYC2 and HY5 [29,34-36,42-44,52,55]. For example, seedlings mutant for *HY5 *show altered balance of auxin and cytokinin signaling and also has decreased expression of two negative regulators of auxin signaling pathways such as AXR2/IAA7 and SLR/IAA14. The functional overlap of light and JA signaling in defence, wound and shade response has been reported [56,57]. *MYC2 *regulates JA responses via differential regulation of an intermediate spectrum of transcription factors with activating or repressing roles. Furthermore, a JA activated MKK3-MPK6 pathway negatively regulates the expression of *MYC2 *[53]. It has been shown that phytochorme deficient *hy1 *mutant seedlings overproduce JA and also display constant expression of JA inducible defense related genes such *VSP1*. The possible reason may be that there is reduction in the total photoactive phytochrome pool in the *hy1 *mutant background and thereby resulting an altered light sensitivity. This may lead to photo-oxidative stress resulting in upregulation of JA synthesis in *hy1 *mutants [50]. The cross talks among multiple signaling pathways occur at the level of intermediate components of the signaling pathways rather than at the receptor level. For example, cross talks between light and JA signaling is mediated by the transcription factor (intermediate component) MYC2/ZBF1. MYC2/ZBF1 works in cryptochrome mediated blue light signaling pathways [29], however *cry1/cry2 *mutants do not have altered JA responses.

It is worth mentioning here that it has earlier been reported that *hy1 *mutants display shorter roots than wild type plants [50]. However, this study does not find such difference in the absence of JA. The apparent discrepancy may be attributed to the developmental stages the observations were made. Whereas Zhai et al., 2007 found the difference at the early seedling stage, this study demonstrates the results of 16-day-old young adult plants, where the altered hypocotyl length was fairly maintained. In this study, our results demonstrate that HY5 and HY1 act additively or synergistically to regulate the JA-induced root-growth-inhibition and expression of JA-responsive genes. Although *hy5 *mutants do not show altered root growth in JA, the JA inducible gene *VSP2 *was upregulated in *hy5 *mutants in the presence of JA. These results indicate a negative regulatory role of HY5 in JA-mediated regulation of *VSP2*. On the other hand, MYC2 which acts as a negative regulator of light signaling, acts as a positive regulator of JA-mediated *VSP2 *expression Figure 5; [35]. Thus, both these transcription factors work in an opposite manner in light and JA signaling pathways.

## Conclusions

This study demonstrates an overlapping function of HY1 with two important transcription factors of light signaling, HY5 and MYC2, in light and JA signaling pathways. The findings in this work will help to better understand the light signaling in Arabidopsis, and the cross talk of light and JA signaling pathways.

## Methods

### Plant materials, growth conditions and generation of double mutants

Arabidopsis (*Arabidopsis thaliana*) seeds were surface sterilized and sown on Murashige and Skoog plates, then kept at 4°C in darkness for 3 to 5 days, and transferred to specific light conditions at 22°C. The intensities of WL and various colour lights (in the light-emitting diode chamber, Q-Beam 3200-A; (Quantum Devices)) used were described in Yadav et al. (2002). For the generation of double mutants such as *hy1 atmyc2*, homozygous *hy1 *(WS) mutant plants were genetically crossed with *atmyc2-1 *(Col-0) homozygous mutant lines. In the F_2 _generation, seedlings were grown in WL (90 *μ*mol m^-2 ^s^-1^) for the identification of *hy1 *homozygous lines, and long hypocotyl *hy1 *mutants were selected and transferred to soil. To determine the genotype of *AtMYC2 *locus, about 40 seedlings from each line were tested by genomic PCR. F_3 _progenies that were homozygous for *atmyc2 *mutant plants were further examined by RT-PCR and considered as *hy1 atmyc2 *double mutants. For measurement of hypocotyl length, ~30 seedlings were used in each genotype. The hypocotyl length measurement was repeated more than twice with similar results.

### Mutant screen and map-based cloning

Ethyl methanesulfonate (EMS)-mutagenized, *hy5KS50 *M2 seeds of *Arabidopsis thaliana *ecotype Wassilewskija (Ws) were grown on MS media under WL conditions and hypocotyl length was compared with that of wild type and *hy5KS50 *mutant lines. Seedlings that showed enhanced and elongated hypocotyl length (as compared to *hy5KS50) *under all light conditions tested were selected and used for further studies. To identify the genetic basis of the EMS mutation in *ehy5*, we isolated the new *ehy5 *mutant, from the *hy5KS50 ehy5 *double mutant background. The double mutant plants were back-crossed successively to wild-type (WS) and the segregated *ehy5 *mutant seedlings in the F_2 _generation was selected and used for further back-crosses with wild type (WS) for four generations (to purify the background mutations in the EMS treated *hy5-ks50 *mutant plants) before physiological and genetic analysis. The *ehy5 *mutant was out-crossed with Wild-type (Col) ecotype, and the mapping population was selected from F_2 _generation. A total of 823 individual F_2 _plants showing the *ehy5 *phenotype were selected for genetic mapping. Genomic DNA was prepared using the protocol described by Edwards et al. (1991). Cleaved amplified polymorphic sequence (CAPS) and simple sequence length polymorphism (SSLP) markers between Col and Ws were used for mapping *EHY5*. For genetic complementation analysis, a genomic fragment containing the entire *HY1*/At2g26670 coding sequence along with its promoter was amplified from the wild-type (Ws) by PCR and the PCR product was digested with HindIII and SmaI and inserted into same sites of modified pBI121 binary vector. The construct obtained was then introduced into *ehy5 (hy1) *mutant plants using *A. tumefaciens*-mediated transformation. Transformants were selected based on their resistance to kanamycin.

### Root growth measurement

Seeds were on MS media in vertical square plates and stratified at 4°C in dark conditions for 4 days to induce uniform germination. The plates were placed vertically in racks, and the seedlings were grown under constant white light conditions (90 *μ*mol m^-2 ^s^-1^) for 16 days. The root length of wild type, single and double mutants was measured. Approximately 25 to 30 seedlings were used for the root length measurement. The experiments were repeated for three times with similar results.

### Root growth response to methyl jasmonate

Seeds of wild type and mutant plants were plated on MS with 15 *μ*M of methyl jasmonate (Sigma) in square plates, after four days of stratification in cold (4°C), plates were placed vertically in racks, and the seedlings were grown under constant white light conditions (90*μ*mol m^-2 ^s^-1^) for 16 days. The root length of wild type, single and double mutants was measured. For determining the VSP2 expression, six-day-old white-light grown seedlings were mock (only MS solution) or JA treated (50 *μ*M JA in MS solution) for 5 hour. After the time period, seedlings were washed with sterile milliQ, excess water was removed with the tissue paper and the tissue was harvested and snap freeze in liquid nitrogen and total RNA was extracted from 100 mg of tissue, using the RNeasy plant mini kit (Quaigen), and cDNA were synthesized from total RNA using titan one-tube RT-PCR system (Roche Applied Science) following the manufacturer's instructions. Real-time PCR analysis of gene expression was carried out by using LightCycler-FastStart DNA Master^-PLUS ^SYBR Green (Roche Applied Science) and was performed using StepOne Real-Time PCR system (ABI). C_T _values of VSP2 were normalized, relative to that of *ACTIN2 *(Internal control).

The following primers were used for the experiment

***VSP2*-FP**: 5' GGCCTTGCATCTTTACCAAAAC 3'

***VSP2*-RP**: 5' GTAGTAGAGTGGATTTGGGAGC 3'

***ACTIN2*-FP**: 5' AAAGGCTTAAAAAGCTGGGG 3'

***ACTIN2*-RP**: 5' GGGACTAAAACGCAAAACGA 3'

### Real-time PCR analysis

Total RNA was extracted from 100 mg of tissue, using the RNeasy plant mini kit (Quaigen), according to manufacturer's protocol. RT-AMV reverse transcriptase (Roche Applied Science) was used for both semi-quantitative RT-PCR and cDNA synthesis. Real-time PCR analysis of gene expression was carried out by using LightCycler-FastStart DNA Master^-PLUS ^SYBR Green (Roche Applied Science) and was performed using Step-one Real-Time PCR system (ABI). C_T _values of *CAB1 *and *RBCS1A *were normalised, relative to that of *ACTIN2 *(Internal control). Real-time PCR was repeated more than thrice and in each biological experiment three technical replicates were used.

The following primers were used for the experiment

***HY1*-FP**: 5' GTGTATCCCTCTTCTCTATTCC 3'

***HY1*-RP**: 5' TCTGAATCCTAGGTCGAGG 3'

***CAB1- *FP**: 5' GTTAACAACAACGCATGGC 3'

***CAB1-*RP**: 5' CCTCTCACACTCACGAAGCA 3'

***RBCS1A*-FP**: 5' TCGGATTCTCAACTGTCTGATG 3'

***RBCS1A*-RP**: 5' ATTTGTAGCCGCATTGTCCT 3'

***ACTIN2*-FP**: 5' TGATGCACTTGTGTGTGACAA 3'

***ACTIN2*-RP**: 5' GGGACTAAAACGCAAAACGA 3'

### Chlorophyll and anthocyanin measurements

Chlorophyll and anthocyanin contents were measured following essentially the same protocols as described in [41]. For chlorophyll and anthocyanin estimation, 50 seedlings were used in each genotype and the experiment was repeated thrice; and in each biological experiment, four technical replicated were used.

## Authors' contributions

VBRP was involved in map-based cloning of EHY1/HY1, generation of double mutants, phenotypic characterization, JA responsiveness and gene expression study. VSK carried out the *hy5 *enhancer screen and identified and partly characterize the *ehy5 *mutant. AN helped in the map-based cloning and participated in the design of the manuscript. SC conceived of the study, participated in its design and coordination, and helped to draft the manuscript. All authors read and approved the final manuscript.
